# The role of GPR1 signaling in mice corpus luteum

**DOI:** 10.1530/JOE-15-0521

**Published:** 2016-07-01

**Authors:** Ya-Li Yang, Li-Rong Ren, Li-Feng Sun, Chen Huang, Tian-Xia Xiao, Bao-Bei Wang, Jie Chen, Brian A Zabel, Peigen Ren, Jian V Zhang

**Affiliations:** 1 Research Laboratory for Reproductive HealthShenzhen Institutes of Advanced Technology, Chinese Academy of Sciences, Shenzhen, China; 2 Shenzhen Key Laboratory of Birth DefectsShenzhen Baoan Maternal and Child Health Hospital, Shenzhen, Guangdong, China; 3 University of Chinese Academy of SciencesShenzhen, China; 4 Laboratory of Immunology and Vascular BiologyDepartment of Pathology, Stanford University School of Medicine, Stanford, California, USA, and Center for Molecular Biology and Medicine, Veterans Affairs Palo Alto Health Care System, Palo Alto, California, USA

**Keywords:** chemerin, GPR1, corpus luteum, progesterone

## Abstract

Chemerin, a chemokine, plays important roles in immune responses, inflammation, adipogenesis, and carbohydrate metabolism. Our recent research has shown that chemerin has an inhibitory effect on hormone secretion from the testis and ovary. However, whether G protein-coupled receptor 1 (GPR1), the active receptor for chemerin, regulates steroidogenesis and luteolysis in the corpus luteum is still unknown. In this study, we established a pregnant mare serum gonadotropin-human chorionic gonadotropin (PMSG-hCG) superovulation model, a prostaglandin F2α (PGF2α) luteolysis model, and follicle and corpus luteum culture models to analyze the role of chemerin signaling through GPR1 in the synthesis and secretion of gonadal hormones during follicular/luteal development and luteolysis. Our results, for the first time, show that chemerin and GPR1 are both differentially expressed in the ovary over the course of the estrous cycle, with highest levels in estrus and metestrus. GPR1 has been localized to granulosa cells, cumulus cells, and the corpus luteum by immunohistochemistry (IHC). *In vitro*, we found that chemerin suppresses hCG-induced progesterone production in cultured follicle and corpus luteum and that this effect is attenuated significantly by anti-GPR1 MAB treatment. Furthermore, when the phosphoinositide 3-kinase (PI3K) pathway was blocked, the attenuating effect of GPR1 MAB was abrogated. Interestingly, PGF2α induces luteolysis through activation of caspase-3, leading to a reduction in progesterone secretion. Treatment with GPR1 MAB blocked the PGF2α effect on caspase-3 expression and progesterone secretion. This study indicates that chemerin/GPR1 signaling directly or indirectly regulates progesterone synthesis and secretion during the processes of follicular development, corpus luteum formation, and PGF2α-induced luteolysis.

## Introduction

Chemerin, a recently discovered adipose cytokine, is also known as tazarotene-induced gene 2 (*TIG2*) and retinoic acid receptor responder protein 2 (RARRES2) (Zabel *et al*. 2006). Initially, chemerin was considered to be a chemoattractant ligand of G protein-coupled receptors (GPRs), but was later found to be an adipocytokine that can regulate fat formation and adipocyte metabolism (Bozaoglu *et al*. 2007). It is secreted from white adipocytes and widely expressed in multiple tissues in the human body, not only in liver and white adipose tissue, but also in placenta, skin, adrenal gland, lung, intestine, pancreas, and ovary (Wittamer *et al*. 2003). The physiological functions of chemerin include regulation of blood pressure, inflammation, immune responses, differentiation of fat cells, and sugar metabolism (Fatima *et al*. 2014), and it plays a key role in metabolic diseases, such as obesity and diabetes (Goralski *et al*. 2007).

Chemerin acts through three receptors, chemokine-like receptor 1 (CMKLR1), G protein-coupled receptor 1 (GPR1), and chemokine (C-C motif) receptor like 2 (CCRL2), as an endocrine, paracrine, and autocrine signaling molecule. All three receptors are seven transmembrane domains (Bondue *et al*. 2011). Chemerin binding to CMKLR1 enhances leukocyte chemotaxis (Peng *et al*. 2015). Chemerin binding to CCRL2 does not stimulate chemotaxis, but might present chemerin to nearby CMKLR1-positive cells to promote its function and play a key role in immune responses, inflammation, and other physiological processes (Zabel *et al*. 2008). While no physiological function has been reported for chemerin binding to GPR1, it has been reported that GPR1 is highly expressed in murine animal brown adipose tissue, white adipose tissue, and skeletal muscle. GPR1 is mainly expressed in vascular cells in white adipose tissue (Rourke *et al*. 2014). In *Gpr1*-knockout mice fed a high-fat diet, glucose intolerance was found to be more serious than in WT mice. Furthermore, in a test of pyruvic acid tolerance, *Gpr1*-knockout mice were able to suppress glucose-stimulated insulin level rise, causing a rise in blood sugar (Nazarko *et al*. 2008). These results suggest that GPR1 is an active receptor of chemerin and that it could regulate glucose homeostasis in the development of obesity.

It was recently reported that many chemokines play important roles in fertility and reproduction (Hausman & Barb 2010). Tena-Sempere *et al*. (1999) found that in rat testicular tissue *in vitro*, leptin inhibited the ground state, and human chorionic gonadotropin (hCG)-stimulated testosterone secretion. Caminos *et al*. (2008) found that after adiponectin treatment, the ground state and hCG-stimulated testosterone secretion by testicular tissue were suppressed. These results suggest that adipose cytokines with endocrine functions have direct and indirect regulatory effects on testosterone secretion by testicular cells. Similarly, many reports have recently focused on the role of the novel chemokine chemerin in the female reproductive system.

Previous research has shown that chemerin inhibits gonad hormone secretion from testis and ovary (Li *et al*. 2014*b*). Studies have shown that the level of chemerin is increased in the blood and adipose tissue of patients with polycystic ovary syndrome (Tan *et al*. 2009). The level of chemerin in the blood of patients with preeclampsia is also increased (Duan *et al*. 2012). Studies have further found that chemerin is expressed in both human and rat placenta and that, in rats, the chemerin level is higher in placenta than in liver (Garces *et al*. 2012). Chemerin and its receptor, CMKLR1, have been reported to be expressed in human granulosa cells. Chemerin treatment can inhibit insulin-like growth factor-induced progesterone and estradiol secretion, and in rat granulosa cells, chemerin can suppress follicle-stimulating hormone (FSH)-induced progesterone and estradiol secretion (Reverchon *et al*. 2012). These results indicate that chemerin can suppress the secretion of gonadal hormones and may be an important factor in obesity and obesity-induced abnormal secretion of gonadal hormones.

The corpus luteum is a transient endocrine organ. During its development, the corpus luteum generates a series of steroids, which cause the corpus luteum to have very high metabolic activity and play an important role in maintaining normal reproductive function in mammals (Pate *et al*. 2012). At the same time, these steroids promote rapid cell growth, proliferation, differentiation, and angiogenesis (Devoto *et al*. 2009).

Chemerin and its receptors are expressed in human and mouse ovary and may suppress sexual hormone secretion (Wang *et al*. 2013), but there are no reports on the relationship between chemerin and the corpus luteum. Our study is the first report on the expression of the novel adipokine chemerin and its receptor GPR1 in mouse corpus luteum and characterization of its direct biological effects on steroidogenesis and luteolysis of the corpus luteum.

## Materials and methods

### Animals

Female C57BL/6 mice (25-day-old) were obtained from Guangdong Medical Laboratory Animal Center. All procedures related to animal use were approved by the Committee on the Use of Live Animals for Teaching and Research, Shenzhen Institutes of Advanced Technology, Chinese Academy of Sciences.

### Determination of estrous cycle stage

For estrous cycle staging, vaginal smears were performed daily in the morning at the same time each day using cotton swabs wetted with PBS; smears were placed on glass slides and cytology was evaluated under a microscope. Estrus was determined by the presence of cornified cells. Metestrus was scored by the presence of large round cells with an irregular border. A high density of leukocytes indicated the stage of diestrus, whereas small nucleated cells indicated proestrus (Becker *et al*. 2005).

### Superovulating corpus luteum model

The 25-day-old immature female mice were injected i.p. with 5IU of pregnant mare serum gonadotropin (PMSG) (ProSpec, Ness-Ziona, Israel) to stimulate follicular development, followed by injection of 5IU of hCG (Sigma) 48h after PMSG injection to induce ovulation.

Animals were anaesthetized before blood were collected by removal of eyeball and then killed by cervical dislocation before the ovaries samples were collected at 24 and 48h after PMSG administration and 24, 48, 72, and 96h after hCG administration. Serum samples were send to Beijing North Institute of Biological Technology (Beijing, China) to measure hormone level of estradiol, progesterone and testosterone. Ovaries were either put in RNAiso Plus (Takara Bio) and stored at −80°C for total RNA isolation or fixed in Bouin’s solution and embedded in paraffin for histological ­examination.

### Postpartum corpus luteum model

Six- to 8-week-old females were housed with males, the occurrence of copulatory plugs was verified by visual examination at each day morning. Females on the third, fourth, and fifth postpartum day were injected s.c. with 25μg/100µL PGF2α, control group injected s.c. with saline. Ovaries and blood samples (collected by removal of eyeball) were taken at 12h after PGF2α and saline injected. Serum was send to Beijing North Institute of Biological Technology to measure hormone level of estradiol, progesterone and testosterone. Ovaries were either snap frozen in RNAiso Plus and stored at −80°C for RNA or fixed in Bouin’s solution and embedded in paraffin for histological examination.

### GPR1 antibody-PGF2α model

Twenty-five-day-old immature female mice were injected i.p. with 1mg/kg mouse GPR1 antibody and 5IU of PMSG, followed by the injection of 1mg/kg mouse GPR1 antibody and 5IU of hCG 48h after PMSG, another group injected 1mg/kg rat IgG as control, 96h after hCG, injected PGF2α to induce luteolysis, another group injected saline as control. Ovaries and blood samples (collected by removal of eyeball) were taken at 6h after PGF2α and saline injected. Serum was send to Beijing North Institute of Biological Technology to measure hormone level of estradiol, progesterone and testosterone. Ovaries were either snap frozen in RNAiso Plus and stored at −80°C for RNA or fixed in Bouin’s solution and embedded in paraffin for histological examination.

### RNA analysis by quantitative PCR

Total RNA from tissues was extracted using RNAiso Plus and subjected to qPCR analysis. RNA samples (0.5μg) were reverse transcribed into cDNA according to the manufacturer’s instructions (Toyobo, Osaka, Japan). The PCR mixtures contained 10μL SYBR Premix Ex Taq II (Toyobo), 1μL of each primer, 1μL cDNA, and 7μL DNase-free water to a final volume of 20μL. Cycle conditions were 10 s at 95°C, followed by 45 cycles at 95°C for 5s, at 60°C for 30s, and at 72°C for 30s. The reaction was completed with a dissociation step for melting point analysis at 50–95°C (in increments of 0.5°C for 10s each). The primers were designed on the basis of the published sequences of *Chemerin* (forward, TGTGCAGTGGGCCTTCCA; reverse, CAAAGGTGCCAGCTGAGAAGA), *Gpr1* (forward, GGAGCTCAGCATTCATCACA; reverse, GACAGGCTCTTGGTTTCAGC), steroidogenic acute regulatory protein (*Star*; forward, CTGCTAGACCAGCCCATGGAC; reverse, TGATTTCCTTGACATTTGGGTTCC), cytochrome P450 cholesterol side-chain cleavage (*P450scc*; forward, CTATGCCATGGGTCGAGAAT; reverse, CAGCACGTTGATGAGGAAGA), 3β-hydroxysteroid dehydrogenase (*Hsd3b*; forward, AGCAAAAAGATGGCCGAGAA; reverse, GGCACAAGTATGCAATGTGCC), and *β-actin* (forward, GGAAATCGTGCGTGACATTA; reverse, AGGAAGGAAGGCTGGAAGAG).

The RNA levels were calculated by 2^−ΔCT^ method, where CT was the cycle threshold (Livak & Schmittgen 2001). The PCR products were confirmed by sequencing. Melting curve analysis for each primer set revealed only one peak for each product, and the sizes of PCR products were confirmed by comparing sizes with a commercial ladder after agarose gel electrophoresis. The results of real-time PCR products were normalized to a stable control, β-actin, which was used as the reference gene.

### Immunohistochemistry

Ovaries from 25-day-old C57BL/6 mice were dissected after decapitation and then fixed, processed for embedding in paraffin, and sectioned. IHC was carried out on 5μm sections of paraffin-embedded tissue. The primary antibodies used for IHC were mouse GPR1 (clone 043, gift from B A Zabel and E Butcher, Stanford University, Stanford, CA, USA), mouse StAR (ab96637, Abcam), and mouse caspase-3 (Abcam), diluted 1:100 in PBS with 1% BSA. The secondary antibodies were horseradish peroxidase (HRP)-donkey-anti-rat (Abcam) to GPR1 and HRP-anti-rabbit (Cell Signaling Technology, Beverly, MA, USA) to caspase 3 and StAR, diluted 1:200 in PBS with 1% BSA. Staining was visualized using a DAB Substrate Kit for peroxidase (Gene Tech, Hyderabad, India), and slides were counterstained with hematoxylin. Control sections were immunostained with a nonspecific IgG to check for nonspecific staining.

### Follicle culture

Twenty-five-day-old immature female mice were injected i.p. with 5IU of PMSG (ProSpec) to stimulate follicular development. Forty-eight hours after PMSG injection, mice were killed, and both ovaries were removed. A 1mL syringe needle was used to remove fat and mesangial tissue around the ovary, and then eye tweezers and needles were used to mechanically isolate follicles under a stereomicroscope. Follicles isolated from the same ovary were added to a single well of a 24-well plate with 1mL DMEM F12, cocultured with 100nM recombinant mouse chemerin (R&D Systems), 0.01IU/mL hCG (Sigma) and 15nM (IC_50_=3nM) phosphoinositide 3-kinase (PI3K) signaling pathway inhibitor wortmannin (Invitrogen). For groups receiving antibody treatment, follicles were precultured for 1–2h with 0.5μg/mL mouse GPR1 antibody (Stanford Brian’s laboratory) before drug added. Other groups added 0.5μg/mL rat IgG (Abcam) as control. Plates were incubated at 37°C, 5% CO_2_ (Li *et al*. 2014*a*), and follicles and media were collected after 6 h. Media were analyzed for hormone levels by the Beijing North Institute of Biological Technology, and follicles were added to RNAiso Plus and stored at −80°C for qPCR detection of related genes.

### Luteal tissue culture

Twenty-five-day-old immature female mice were injected i.p. with 5IU of PMSG (ProSpec) to stimulate follicular development, followed by injection of 5IU of hCG (Sigma) 48h after PMSG injection to induce ovulation. Seventy-two hours after hCG injection, mice werekilled, ovaries were removed, fat and mesangial tissue were removed from around the ovary using a 1mL syringe needle, and luteal tissue was mechanically separated under a stereomicroscope using eye tweezers and needles. Luteal tissue isolated from the same ovary were added to a single well of a 24-well plate with 1mL DMEM F12, cocultured with 100nM recombinant mouse chemerin (R&D Systems) and 0.01IU/mL hCG (Sigma). For groups receiving antibody treatment, follicles were precultured for 1–2h with 0.5μg/mL mouse GPR1 antibody (Stanford Brian’s laboratory) before adding drug. Other groups added 0.5μg/mL rat IgG (Abcam) as control. Plates were incubated at 37°C, 5% CO_2_, and luteal tissue and media were collected after 24h. Media were analyzed for hormone levels by the Beijing North Biotechnology Research Institute and luteal tissue was placed in RNAiso Plus and stored at −80°C for qPCR detection.

### Hormone measurements by RIA

Progesterone and estradiol levels in conditioned media were measured using commercial iodine [^125^I] RIA Kits (Beijing North Biotechnology Research Institute). The sensitivity of the progesterone and estradiol RIA assays was 20ng/mL. The intra-assay error and inter-assay error were <10 and <15%, respectively.

### Statistical analysis

All data are presented as mean±s.e.m. and statistical significance was assessed by either one-way ANOVA followed by Fisher’s least significant difference test for *post hoc* comparisons or the Student’s *t*-test (GraphPad Prism). A *P* value of <0.05 was considered to be statistically significant.

## Results

### Expression of *Chemerin* and *Gpr1* in mouse ovary during the estrous cycle

**Figure 1:**
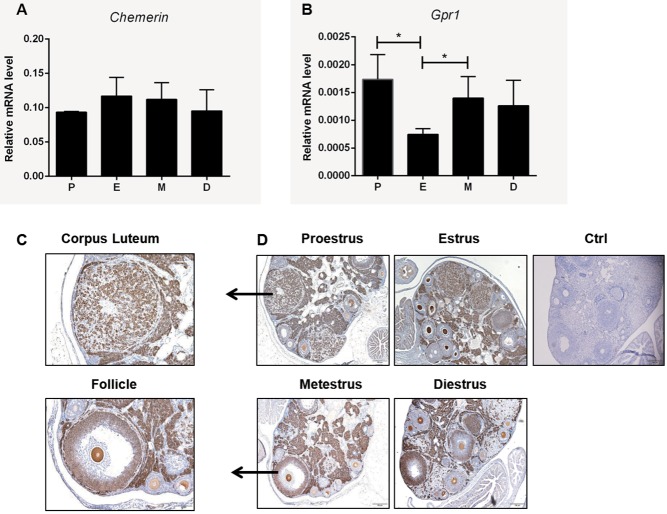
Expression of *Chemerin* and *Gpr1* in mouse ovary during the estrous cycle. (A) *Chemerin* mRNA expression was measured by qPCR analysis of ovaries from 8- to 12-week-old female mice, *N*≥3. (B) *Gpr1* mRNA expression was measured by qPCR analysis of ovaries from 8- to 12-week-old female mice, *N*≥3; Student’s *t*-test, *P<0.05 as indicated. (C) Immunolocalization of GPR1 in corpus luteum and follicles. Scale bars=50αm. (D) Immunolocalization of GPR1 in mouse ovary during the estrous cycle. Parallel sections are immunostained with nonimmune serum as a control; scale bars=100αm.

*Chemerin* and *Gpr1* mRNAs were found to be expressed in mouse ovary during the estrous cycle (Fig. 1A and B), suggesting that chemerin and GPR1 play direct or indirect roles in the regulation of follicle and corpus luteum development. IHC staining showed that, at various stages of the estrous cycle, *Gpr1* was expressed at high levels in developing follicles at all stages of development and in the stroma, mainly in thecal cells, granulosa cells, luteal cells, and interstitial cells (Fig. 1C and D). Interestingly, G*pr1* staining in the follicle appears to be mostly in the oocytes, and absent in the granulosal cells of all except the tertiary follicles, which could be studied furthermore. These results indicate that the chemerin/GPR1 signaling pathway plays an important role in follicular development and corpus luteum formation.

### In a mouse superovulation model, chemerin can suppress hCG-stimulated progesterone production in follicle and luteal tissue cultures

**Figure 2:**
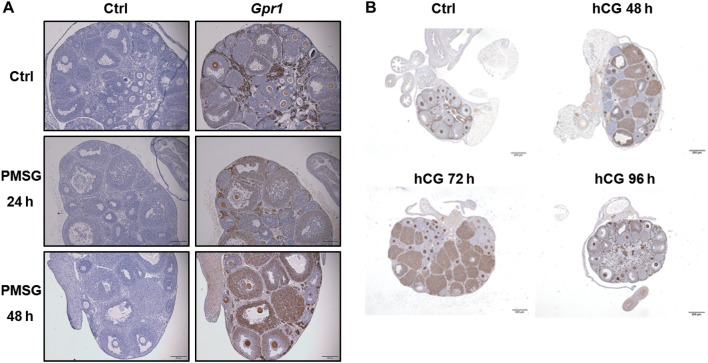
Immunolocalization of *Gpr1* in mouse ovary during superovulation. (A) Immunolocalization of GPR1 in mouse ovary after i.p. PMSG; in this phase, follicles develop and mature. Scale bars=100μm. (B) Immunolocalization of GPR1 in mouse ovary after i.p. hCG; in this phase, ovulation occurs, followed by corpus luteum formation and regression. Parallel sections were immunostained with nonimmune serum as a control; scale bars=1mm.

*Gpr1* is highly expressed in the oocytes, interstitial tissue, granulosa cells, and theca cells, specifically in the corpus luteum of superovulated mouse ovaries. IHC staining showed that, on the second day after PMSG injection, follicles were either mature or in the process of ovulation, and on the third day after hCG injection, the number and size of corpus lutea was at their greatest (Fig. 2).

**Figure 3:**
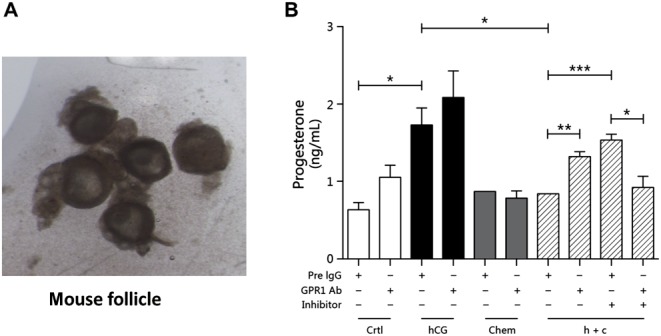
Suppressive effect of chemerin on progesterone in mouse follicles in an *in vitro* model. (A) Dissected antral follicle. (B) Progesterone levels in cultured follicles with different treatments. Inhibitor, PI3K inhibitor wortmannin; Chem, chemerin; h+c, hCG+chemerin. Each experiment was performed at least three times; all data are presented as mean±s.e.m.; *P<0.05, **P<0.01, ***P<0.005 for one-way ANOVA followed by Fisher’s least significant difference test.

Antral follicles were isolated under a stereomicroscope. Different drug treatments had different effects on progesterone secretion. After hCG treatment, progesterone levels increased substantially, indicating that hCG could promote the secretion of progesterone. After chemerin cotreatment with hCG, the progesterone level increase was significantly attenuated, indicating that chemerin may suppress progesterone secretion. Interestingly, when a PI3K signaling pathway inhibitor was added, the suppressive effect of chemerin almost disappeared, suggesting that chemerin suppresses progesterone secretion through PI3K signaling. Furthermore, in the presence of both chemerin and hCG, the suppressive effect of chemerin was reduced by GPR1 MAB treatment, indicating that GPR1 is the active receptor acting through the PI3K pathway to mediate chemerin’s effect on progesterone secretion (Fig. 3).

**Figure 4:**
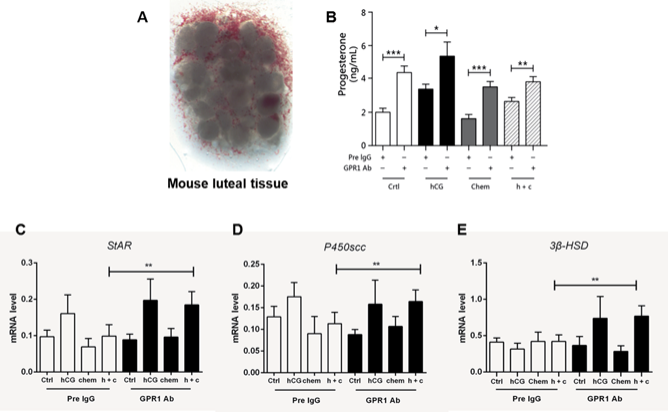
Suppressive effect of chemerin on progesterone in luteal tissue in an *in vitro* model. (A) Dissected mouse luteal tissue. (B) Progesterone levels in cultured luteal tissue with different treatments. Chem, chemerin; h+c, hCG+chemerin. Each experiment was performed at least three times. (C) Relative gene expression of *Star* in cultured luteal tissue. (D) Relative gene expression of *P450scc* in cultured luteal tissue. (E) Relative gene expression of *3β-HSD* in cultured luteal tissue. β-actin served as the reference gene; *P<0.05, **P<0.01, ***P<0.005 for one-way ANOVA followed by Fisher’s least significant difference test; *n*≥3, all data are presented as mean ± s.e.m.

Luteal tissue from superovulating ovaries was isolated under a stereomicroscope (Fig. 4A). As observed for follicle culture, different drug treatments had different influences on progesterone secretion. For the pre-IgG control group, after hCG treatment, progesterone levels increased remarkably, whereas with further addition of chemerin, the progesterone increase was significantly inhibited. For the GPR1 antibody treatment group, the pattern was same as that for the control group, except that progesterone levels were higher under all conditions (Fig. 4B). In addition, hCG stimulation resulted in the upregulation of key steroidogenic factors, such as *Star, P450scc,* and *3β-Hsd*. The suppressive effect of chemerin on hCG-stimulated progesterone production was accompanied by the suppression of hCG-stimulated expression of *Star, P450scc*, and *3β-Hsd.* When anti-GPR1 antibody was added, the suppressive effect disappeared (Fig. 4C, D and E).

From the above results, it can be inferred that chemerin could suppress progesterone secretion through its receptor, GPR1, during ovarian follicle development and in the process of corpus luteum formation.

### In postpartum corpus luteum, PGF2α could induce luteolysis while GPR1 expression decreases

**Figure 5:**
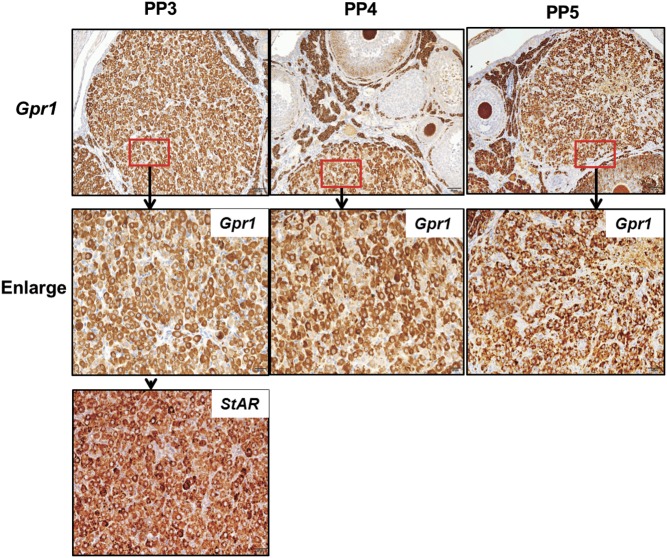
Immunolocalization of *Gpr1* and *StAR* in postpartum mouse ovary. PP3, third-day postpartum; PP4, fourth-day postpartum; PP5, fifth-day postpartum. Parallel sections were immunostained with nonimmune serum as a control; scale bars=50μm. Enlarged images of corpus luteum are shown in the lower panels. scale bars=20μm.

The superovulation model is based on the use of immature female mice treated with hormones to artificially induce maturation and ovulation, leading to corpus luteum formation. In order to study the role of chemerin/GPR1 signaling in the development of ovarian follicles and the corpus luteum, we established a natural mature corpus luteum model. On the third to fifth days postpartum, immunohistochemical results showed that *Gpr1* was still highly and specifically expressed in the corpus luteum with positive staining for StAR. On the third day after birth in mice, the structure of corpus luteum was still complete and had not been regressed (Fig. 5), so we chose the third day postpartum to establish the PGF2A-induced luteolysis model in mice.

**Figure 6:**
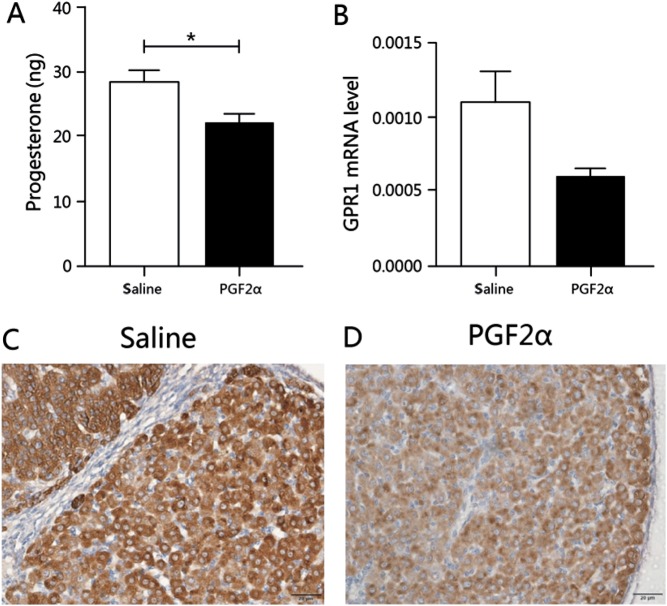
*Gpr1* expression decreases in PGF2α-induced postpartum luteolysis. (A) Serum progesterone levels in saline control- and PGF2α-injected mice. (B) *Gpr1* mRNA levels in the ovaries of control- and PGF2α-injected mice. (C) Immunolocalization of *Gpr1* in the saline-injected control group. (D) Immunolocalization of *Gpr1* in the PGF2α-injected group. Scale bars=20μm. β-actin served as the reference gene; *n*≥3, all data are presented as mean ± s.e.m.; *P<0.05 for the Student’s *t*-test.

In the PGF2α-induced luteolysis model, first, compared with the control group, serum progesterone levels in the PGF2α injection group decreased significantly (Fig. 6A), and corpus luteum structure was relatively regressed compared with that in the control group. qPCR showed that *Gpr1* mRNA levels in the PGF2α-injected ovary have a declined tendency relative to levels in the control group, but there was no significant difference (Fig. 6B). IHC staining with anti-GPR1 antibody produced a weaker signal in the PGF2α-treated corpus luteum than in the corpus lutea from the control group (Fig. 6C and D), suggesting that the expression of *Gpr1* declines during the process of PGF2α-induced luteolysis.

### In superovulating corpus luteum, GPR1 antibody suppresses PGF2α-induced luteolysis

**Figure 7:**
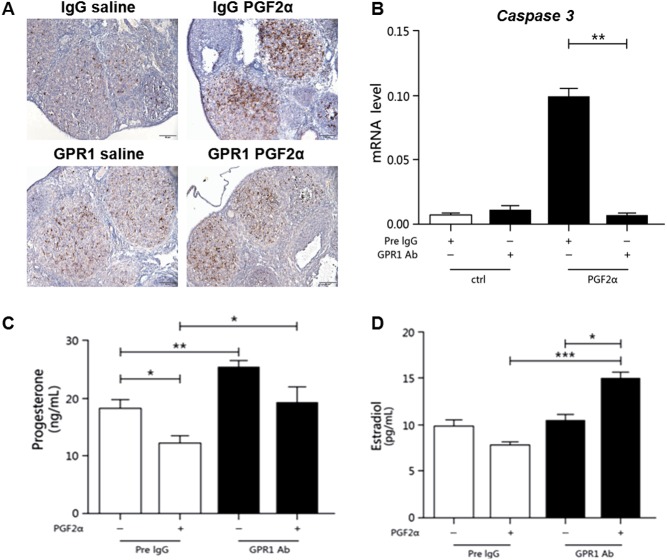
*Caspase-3* expression and serum hormone levels in the PGF2α *in vivo* model. (A) Immunolocalization of *Caspase-3* in GPR1 antibody injection model mouse ovaries; scale bars=50 µm. (B) *Caspase-3* mRNA levels in GPR1 antibody injection model mouse ovaries. (C) Serum progesterone levels in the GPR1 antibody injection model. (D) Serum estradiol levels in the GPR1 antibody injection model; *n*≥3, *P<0.05, **P<0.01, ***P<0.005 for one-way ANOVA followed by Fisher’s least significant difference test. All data are presented as mean±s.e.m.

GPR1 antibody was injected into 25-day-old mice undergoing PMSG-hCG-induced superovulation, while pre-immune IgG injection served as a control. On the second day after hCG injection, PGF2α was administrated to induce luteolysis. The levels of *Caspase-3* mRNA in the GPR1 antibody-injected group was significantly reduced compared with levels in the control group after PGF2α injection (Fig. 7B). Furthermore, immunolocalization of *Caspase-3* showed that the number of apoptotic luteal cells was significantly reduced in the GPR1 antibody-injected group (Fig. 7A).

After GPR1 antibody treatment, serum progesterone levels rose significantly, in accordance with the follicle and luteal tissue culture results. After PGF2α injection, progesterone levels decreased significantly, but serum progesterone levels were significantly higher in the mice with GPR1 antibody administration than in the pre-IgG control group (Fig. 7C). The trend in serum estradiol levels was roughly the same as that for progesterone (Fig. 7D). These results showed that, after blocking the function of GPR1, PGF2α-induced apoptosis and hormone secretion in the corpus luteum was suppressed, indicating that chemerin signaling is mediated by GPR1 and plays a role in promoting luteolysis.

## Discussion

Follicular development directly influences the number of ova and ovarian endocrine functions, such as secretion of steroid hormones (estrogen, progesterone, and testosterone) (Su & Eppig 2002). Theca cells, granulosa cells, and oocytes are the three main ovarian cells. Theca cells secrete androgen; granulosa cells use androgen to synthesize estrogen and can also secrete progesterone independently. The synthetic process is regulated by a series of steroid synthetases, including StAR, P450scc, 3β-HSD, and 17β-HSD (Liu & Hsueh 1986).

Schipper *et al*. (1993) found that FSH and luteinizing hormone (LH) can increase the levels of progesterone and estrogen in granulosa cells exponentially using *in vitro* cultured human ovarian granulosa cells. Some cytokines also have an effect on granulosa cells: insulin-like growth factor (IGF) can promote estrogen secretion by granulosa cells (Monniaux & Pisselet 1992); TNF-α can inhibit FSH stimulation of granulosa cell aromatase activity, and can suppress LH, resulting in a reduction in androgen production (Zolti *et al*. 1991); interleukin 1 (IL-1) can reduce the ability of granulosa cells to synthesize progesterone, and reduce luteinizing hormone receptor (LHR) in granular cells (Rivier & Vale 1989). Recent studies have demonstrated that resistin is expressed in pig granulosa cells and that this also increases follicle progesterone and testosterone synthesis and secretion by increasing the expression of *CYP11A1, 3β-HSD, CYP17A1, 17β-HSD*, and *CYP19A1* (Sirotkin *et al*. 2001).

**Figure 8:**
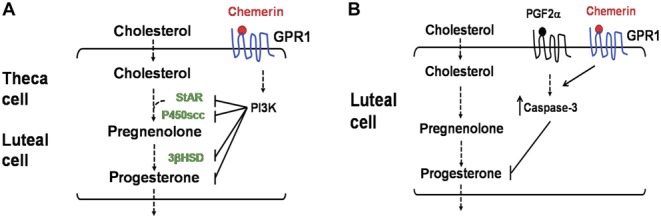
Chemerin/GPR1 signaling suppresses progesterone synthesis and secretion during follicle/corpus luteum development and luteolysis in mice. (A) Chemerin could suppress progesterone synthesis through its receptor GPR1 during follicle and corpus luteum development in mice, the process go through PI3K pathway and effect some key enzymes such as StAR, P450scc, and 3-HSD in the synthesis of progesterone. (B) PGF2α lead to caspase-3, highly expressed, further induced luteolysis and progesterone level significantly decreased. Chemerin cooperate with this process through its receptor GPR1.

In our study, we found that *chemerin* and its receptor, *Gpr1*, were highly expressed in ovaries in multiple stages of the estrous cycle, we infer that chemerin and GPR1 may have regulatory effects on the processes of follicular development and corpus luteum formation. IHC results showed that *Gpr1* was localized to follicular granulosa cells, theca cells, and the cumulus oophorus. The staining in the follicle appears to be mostly in the oocytes, and absent in the granulosal cells of all except the tertiary follicles. Based on this interesting finding we have two types of hypotheses, the first is that GPR1 may be involved in oocyte growth and maturation, the second is that the expression of GPR1 on granulosa cell or theca cell may associate with the formation of mature follicle, while we need to do more research to confirm our hypothesis. *In vitro* follicle culture indicated that chemerin suppresses hCG-induced progesterone secretion by follicles at a concentration of 100 nM, whereas when GPR1 antibody was added, the suppressive effect of chemerin weakened, and when a PI3K signaling inhibitor was added, the inhibitory effect of chemerin was abrogated (Fig. 8A). From these results, we speculate that the chemerin/GPR1 and PI3K signaling pathways are involved in processes associated with follicular development, such as progesterone production and secretion.

The corpus luteum is a temporary endocrine organ that plays an important role in the process of female reproductive cycle regulation and maintenance of pregnancy; luteal phase defect (LPD) can cause female infertility or first trimester miscarriage (Wuttke *et al*. 2001).

The corpus luteum is formed by rupture of follicles after ovulation and is associated with rapid blood vessel growth (Reynolds *et al*. 2003). Its main physiological function is to secrete steroid hormones, mainly ­progesterone, but also androgen and estradiol. Androgen stimulates the secretion of estrogen and progesterone, while the most important function of estradiol is to promote the formation of blood vessels in the middle of pregnancy (­Skarzynski *et al*. 2008). Formation of early luteal blood vessels plays a very important role in the development of the corpus luteum. At present, many studies have indicated that vascular endothelial growth factor (VEGF) plays a central role in the formation and evolution of the corpus luteum (Duncan *et al*. 2008). Adipose cytokines have also been reported in the process of corpus luteum development. Nicklin *et al*. (2007) found that leptin also had a positive effect on the establishment of the corpus luteum.

In our study, *Gpr1* was found to be highly expressed in the corpus luteum, while chemerin was found to ­suppress hCG-induced progesterone secretion by the corpus luteum at a concentration of 100 nM, and the suppression was accompanied by the inhibition of *Star* and *P450scc* expression. With the addition of GPR1 antibody, the suppressive effect of chemerin weakened, and the expression of *Star* and *P450scc* recovered (Fig. 8A). This means that the chemerin/GPR1 signaling pathway plays an important role in the development of the corpus luteum.

In order to maintain normal reproductive function, without fertilization or pregnancy failure, luteolysis occurs. In rodents, luteolysis has two stages: the first stage is functional degradation, when progesterone levels drop significantly, while the second stage is structural degradation, when there is programed death of luteal cells (­Xuejing *et al*. 2013). These processes are affected by many factors (Stocco *et al*. 2007), with PGF2α playing an important role.

By inhibiting cholesterol transport (Pescador *et al*. 1996), side chain rupture (Murdoch *et al*. 1996), and the stimulation of progesterone synthesis by gonadotropin (Bjurulf *et al*. 1998), inhibiting the release of progesterone (Hayashi & Miyamoto 1999) and reducing the concentration of progesterone in serum and in the corpus luteum, PGF2α causes corpus luteum dissolution. Damage to luteal cell membranes and the induction of luteal cell apoptosis (Bo *et al*. 1999) lead to corpus luteum structural degradation, involving in a variety of cytokines and immune functions.

In our PGF2α-induced luteolysis mouse model, expression of *Gpr1* and serum progesterone levels decreased, and *Caspase-3* expression increased. While the addition of GPR1 antibody elevated serum progesterone levels, expression of *Caspase-3* declined significantly, and PGF2α-induced luteolysis was suppressed (Fig. 8B). We conclude that chemerin participates in the process of luteolysis through GPR1.

In conclusion, chemerin/GPR1 signaling was found to play an important role in follicular development, corpus luteum development, and luteolysis and may suppress progesterone secretion and promote PGF2α-induced luteolysis. The effect of chemerin and GPR1 on steroidogenesis in the corpus luteum and luteolysis will potentially lead to therapeutic interventions into infertility caused by LPD.

## Declaration of interest

The authors declare that there is no conflict of interest that could be perceived as prejudicing the impartiality of the research reported.

## Funding

This work was supported by the National Major Basic Research Program of China (2013CB945503) to J V Z, Guangdong Grant 2015A020212030 to P G R, Shenzhen Grant (KQCX20140521115045442 to J Z, ZDSYS201504301707152 to L K X, JCYJ20150403105513698 to L R R, and JCYJ20150401150223631 to P G R).

## Author contribution statement

J V Z and Y L Y conceived and designed the experiments; C H, B B W, J C, and T X X performed the experiments; Y L Y, L F S, and L R R analyzed the data; L R R, B A Z, and P G R contributed reagents/materials/analysis tools/housing animals; Y L Y, J V Z, and P G R wrote and revised the manuscript.
